# List of Reviewers: 1st November 2023–31st October 2024

**DOI:** 10.1017/jns.2025.3

**Published:** 2025-05-22

The Editorial Board of *Journal of Nutritional Science* would like to thank the following for their contribution as peer reviewers in 2024:

Bedilu Abebe Abate

Hazreen Abdul Majid

Seham Mohammad Abu Jadayil

Baha’a Mohammad Abu Salma

Peter Acire

Riho Adachi

Marcia Barbosa Aguila

Md. Sabbir Ahmed

Umar Akram

Fahmida Akter

Rafael Almendra-Pegueros

Maria Alvim

Mary Amoako

Anchamo Anato

Lauri Andress

Erik André

Steve D. Anton

Arheiam Arheiam

Gholamreza Askari

Mariam Assaad

Fernanda Souza de Oliveira Assis

Erni Astutik

Mutamed Ayyash

Fusta Azupogo

Bojlul Bahar

Habiba Bajit

Kate Balestracci

Kondwani-Joseph Banda

Sujan Banik

David Barney

Kristine Beaulieu

Robel Sahilu Bekele

Jonathan Bennett

David Benton

Eric P. Berg

Anteneh Berhane

Angela Bermudez-Millan

Sarah E.E. Berry

Shilpa Bhaise

Subir Biswas

Anne Ahrendt Bjerregaard

Jorge L Ble-Castillo

Debra Boardley

Eyob Ketema Bogale

Francisco Bolaños-Jimenez

Venessa Botha

Lana Angélica Braudes Silva

Md Mofijul Islam Bulbul

Natasha Bunzi

Xuan Cai

Philip C. Calder

Francesco Campa

Monica Hauger Carlsen

Natane Carvalho

Sherifath Chabi

Hongxia Chen

Sanmei Chen

Xiaolian Chen

Zhonghua Chen

Matthew Chrisman

Anthea Christoforou

Minjie Chu

Cristiane Cominetti

Kristen Copeland

Sarah Cottin

Marie Cyrenne-Dussault

Christine Dalgård

Savana Daneghian

Shaonong Dang

A. Daniel

Hassan Dashti

Erna Davidović Cvetko

Ali Dehghani

Beruk Berhanu Desalegn

Navin Kumar Devaraj

Neda Dolatkhah

David Doledec

Ruben Abraham Dominguez

Perez

Honglin Dong

Samuel Duran

Johanna T. Dwyer

Zeynep Bengisu Ejder

Ahmed El-Sohemy

Basma Ellahi

Pauline M. Emmett

Silvia Ezemenahi

Ngozika E. Ezinne

Neda Ezzeddin

Amir Hossein Faghfouri

Awat Feizi

Fentaw Wassie Feleke

Jeane Ferreira

Roger Figueroa

Teshale Fikadu

Matt Fossey

Lucine Francis

Mara Anna Franke

Whitney Fung Uy

Theophilus Sunday Gabriel

Xianli Gao

Anna Gawedzka

Ribka Dinku Gebre

Tsegu Gebru

Habtamu Fekadu Gemede

Leyna Germana

Eskezyiaw Agedew Getahun

Tamirat Gezahegn

Umesh Ghimire

Barbara Goldman

Alicia Gomes

Javier Thomas Gonzalez

Damodara K.M. Gowda

Clint Gray

Adyya Gupta

Dominika Guzek

Amir Hadi

W. Mekonnen Haileselassie

Megan L. Hammersley

Fei Han

Mohammmad Hashem

Hashempur

Saima Hasnin

Habtamu Hassen

Qi-Qiang He

Ehsan Hejazi

Catrin Herpich

Oscar Fernando Herran

Javad Heshmati

Allison Hodge

Michael F. Holick

Ryota Hosomi

Somayeh Hosseinpour-Niazi

Mahdieh Hosseinzadeh

Dragan Hrncic

Adela Hruby

Kuen-Chang Hsieh

Temitope Ibiyemi

Annette Imohe

Rabia Javed

Dawit Jember Tesfaye

Mica Jenkins

Baoren Jian

Yannan (Jessica) Jin

William Joe

Robert Johnston

Rajeev Kumar

Niina Eerika Kaartinen

Emma Kalk

Sangeeta Kansal

Majid Karandish

Saeed Karim

Ozgur Kasapcopur

Andargachew Kassa

Kendra Kattelmann

Tsehay Kebede

Marko Kerac

Fariborz Khajali

Manouchehr Khazandi

Sharon I. Kirkpatrick

Soressa Merrera Kitessa

Juergen Koenig

Ezgi Kolay

Hamed Kord-Varkaneh

Tevfik Koçak

Emine Koçyiğit

Tony Kuo

Alparslan Kurtul

Lai Lee Lee

Jeyaseelan Lakshmanan

N. Landy

Ginny Lane

Cristina Lanorio

Vania Aparecida Leandro-Merhi

Lise Leblay

Natasha Lelijveld

Rachel Liebe

Ruvini Liyanage

Esther Lopez-Bayghen

Esther Lopez-Garcia

Mary-Jon Ludy

Victor Mogre

Fernanda Pons Madruga

Emmanuella Magriplis

Rachel Mahas

Rubina Mandlik

Kelsey M. Mangano

Evangeline Mantzioris

Anthony Manyara

Paulo Cezar de Freitas Mathias

Mai Matsumoto

Xikombiso Mbhenyane

Amanda C. McClain

Agerie Mengistie Zeleke

Celine Miani

Catherine Milte

Kyoko Miura

Bekri Mohammed

Shemsu Kedir Mohammed

Temesgen Mohammed

Ahmed E. Abdel Moneim

Sajjad Moradi

Nancy Engelmann Moran

Theodora Mouratidou

Annabel Sandra Mueller-Stierlin

Mirthe Muilwijk

Michelle M. Murphy

Vali Musazadeh

Marta Muszalik

Aimen Nadeem

Zain Ali Nadeem

Mieko Nakamura

Adrian Neagu

Elizabeth P. Neale

Ilana Nogueira Bezerra

Hilda Nyambe Silavwe

Adedayo Ojo

Akinkunmi Okekunle

Vahidreza Ostadmohammadi

Silvia Giselle Ibarra Ozcariz

V. Padmanabhan

Zamzam Paknahad

Manoranjan Pal

Indira Paz Graniel

Marcela Radtke

Pegah Rafiee

Sachchida Nand Rai

Urvashi Rana

Amit Ranjan

Marla M. Reicks

Sofia Reis Alves Soares Cardoso

Yougang Ren

Mahshid Rezaei

Karla Danielly da S. Ribeiro

Brenda Robles

Ana Rodriguez-Mateos

Violeta Magdalena Rojas Huayta

Rajshri Roy

Adriana Rusu

Zoya Sabir

Majid Sadeghpour

Seyedhassan Sadrian

Sevda Saleh-Ghadimi

Codjoe Samuel

Joao Gabriel Sanchez

Elizabeth Sanderman

Ursula Schwab

Manuela Sellitto

Y Selvamani

Mark Sewart

Mohammad Shannawaz

Amrollah Sharifi

Sukshma Srinath Sharma

Hojjat Sheikhbardsiri

Adam Shoesmith

Jitendra Kumar Singh

Taryn J Smith

Jakub Sobiecki

Nelia Patricia Steyn

Alice Stiletto

Barbara J. Stoecker

Norio Sugawara

Koki Sugimoto

Boyd Swinburn

Ahmad Syauqy

Mika Tada

M. Taheri

Enyew Talie

Yanguo Tan

Bryan Tang

Maryam Tarameshlu

Paulo de Mello Tavares Lima

Kidus Temesgen

Norman Temple

Mesfin Wogayehu Tenagashaw

Ana Elisa Toscano

Angeliki Tsapara

Fotini Tsofliou

Hande Gül Ulusoy Gezer

Farhad Vahid

Sabaghi Vahide

Mahdi Vajdi

Rodrigo Valenzuela

Elizabeth Valoyes Bejarano

Tertia Van Zyl

Carina Venter

Swati Verma

Francesco Visioli

Marcia Regina Vitolo

Leigh C. Ward

Marianna Wetherill

Kathryn Whyte

Bethany Williams

Janet Williams

Benjamin P. Willing

Janelle Windus

Renate M. Winkels

Alexei Wong

Charlotte M. Wright

Keke Xiao

Pengcheng Xun

Nurcan Yabancı Ayhan

Najat Yahia

Owusua Yamoah

Wai Yew Yang

Tamiru Yazew

Achileas Zacharoulis

Maham Zahid

Syeda Mahvish Zahra

Meysam Zarezadeh

Guoha Zeng

Jianjun Zhang

Liping Zhang

Tianyi Zhang

Yingze Zhang

Yumeng Zhang

Xiaoyun Zheng

Yanmin Zhou

Muhammad Zia

Khaled Abu Ruz

Osama Albasheer

Sonia de Pascual-Teresa

Rastegar Hoseini

Cindy Taggart

Tess van Rijssel

Emmy van den Heuvel

Menşure Nur Çelik

Büşra Özyalçın

